# Foodomics: A Data-Driven Approach to Revolutionize Nutrition and Sustainable Diets

**DOI:** 10.3389/fnut.2022.874312

**Published:** 2022-05-03

**Authors:** Selena Ahmed, John de la Parra, Ismahane Elouafi, Bruce German, Andy Jarvis, Vincent Lal, Anna Lartey, T. Longvah, Carlos Malpica, Natalia Vázquez-Manjarrez, Jessica Prenni, Carlos A. Aguilar-Salinas, Warangkana Srichamnong, Maya Rajasekharan, Tracy Shafizadeh, Justin Bloomfield Siegel, Roy Steiner, Joe Tohme, Steve Watkins

**Affiliations:** ^1^American Heart Association, Inc., Dallas, TX, United States; ^2^Department of Health and Human Development, Montana State University, Bozeman, MT, United States; ^3^The Rockefeller Foundation, New York, NY, United States; ^4^Harvard University, Cambridge, MA, United States; ^5^Food and Agriculture Organization of the United Nations, Rome, Italy; ^6^Food Science and Technology, University of California, Davis, Davis, CA, United States; ^7^International Center for Tropical Agriculture, Cali, Colombia; ^8^The Institute of Applied Sciences, The University of the South Pacific, Suva, Fiji; ^9^Nutrition and Food Science, University of Ghana, Accra, Ghana; ^10^National Institute of Nutrition, Hyderabad, India; ^11^MLP Vision Biotech SL, Majadahonda, Spain; ^12^National Institute of Nutrition and Medical Sciences, Salvador Zubiran, Mexico City, Mexico; ^13^Horticulture and Landscape Architecture, Colorado State University, Fort Collins, CO, United States; ^14^Institute of Nutrition, Mahidol University, Salaya, Thailand; ^15^Verso Biosciences, Inc., Davis, CA, United States

**Keywords:** food analysis, food composition, food biomolecules, omics technology, mass spectrometry

## Abstract

Globally, we are failing to meet numerous nutritional, health, and environmental targets linked to food. Defining food composition in its full chemical and quantitative diversity is central to data-driven decision making for supporting nutrition and sustainable diets. “Foodomics”—the application of omics-technology to characterize and quantify biomolecules to improve wellbeing—has the potential to comprehensively elucidate what is in food, how this composition varies across the food system, and how diet composition as an ensemble of foods guides outcomes for nutrition, health, and sustainability. Here, we outline: (i) challenges of evaluating food composition; (ii) state-of-the-art omics technology and innovations for the analysis of food; and (iii) application of foodomics as a complementary data-driven approach to revolutionize nutrition and sustainable diets. Featuring efforts of the Periodic Table of Food Initiative, a participatory effort to create a globally shared foodomics platform, we conclude with recommendations to accelerate foodomics in ways that strengthen the capacity of scientists and benefit all people.

## Introduction

Globally, we are failing to meet numerous nutritional, health, and environmental targets linked to food. Unacceptable levels of malnutrition in all its forms (undernutrition, micronutrient deficiency, and overweight and obesity) persist in every country, inequitably affecting the most vulnerable (1). Poor diets are responsible for one in five deaths globally, more than any other risk factor (2). While poor diets vary, they are crudely characterized by low intake of whole grains, fruits, nuts, and seeds, and vegetables and, in some contexts, as having excess intake of sugars (3), saturated fats, calories, and highly processed foods of non-nutritive substances (4, 5). Dietary challenges have compounded with the homogenization of food supplies over the past eight decades (6–8).

As a threat multiplier, malnutrition perpetuates ill-health and cycles of poverty. Poor diets are associated with reduced educational outcomes and labor productivity (2). Concurrently, unsustainable agricultural practices, food-based greenhouse gas emissions, market disruptions, food distribution, and waste, and a growing population are degrading the natural resource base that supports nutrition and food security (9–11). Overall, poor diets impede achieving multiple sustainable development goals (12).

Food composition databases emerged in the 1900s as an essential public health tool to combat malnutrition. Tremendous progress on food composition evaluation has occurred since the 1895 publication of the United States Department of Agriculture (USDA) bulletin featuring Wilbur O. Atwater’s data on food composition and nutritional needs (13). Numerous food composition database efforts, including those of the USDA’s FoodData Central and the International Network of Food Data Systems (INFOODS) administered by the Food and Agriculture Organization (FAO) of the United Nations, have led efforts for multiple decades to advance guidelines, tools, and harmonization of data on food and nutrient profiles for the global community. As the science of human nutrition has evolved from consideration of foods simply as sources of energy and essential nutrients, to recognizing the role of dietary bioactive components to modulate metabolic processes and reduce disease risk (14) within the context of sustainable diets and food systems, food composition data also needs to continue to evolve. Phenol-Explorer (15), Carotenoids Database (16), PhytoHub (17), and FoodDB (18) have emerged to offer food composition data focused on dietary bioactive components.

**FIGURE 1:**
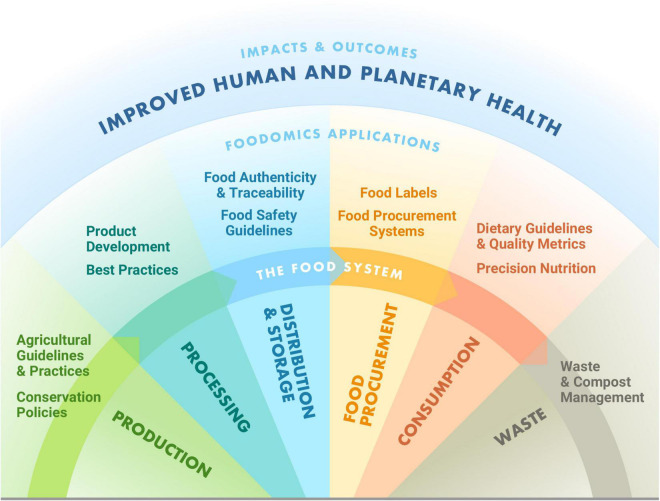
Application of foodomics across the food system. Foodomics can be applied across the food system to enhance human and planetary health.

Foodomics, or the application of omics-technology to characterize and quantify biomolecules in food to explicitly improve wellbeing (19), has the potential to meet food composition needs with unprecedented knowledge (14, 20–23). In a post-genome biology era, advances in analytical chemistry, omics technology (approaches that enable a global assessment of a set of molecules), and computational science provide an enabling environment to address technical challenges of evaluating food composition (21). Innovations in foodomics can complement existing approaches to drive a revolution where we can apply knowledge of food composition across food systems to advance nutrition, health, and environmental targets (Figure 1).

This Perspective outlines: (i) challenges of evaluating food composition; (ii) state-of-the-art omics; and; (iii) application of foodomics. Featuring efforts of the Periodic Table of Food Initiative (PTFI), a participatory effort to create a globally shared foodomics platform, we conclude with recommendations to accelerate foodomics as a complement to other food and dietary approaches in ways that benefit people and the planet.

## Challenges of Evaluating Food Composition for Data-Driven Decision Making

Three key technical challenges limit the potential of evaluating food composition: (i) reproducibility and standardization; (ii) representation and quantification; and (iii) accessibility.

### Reproducibility and Standardization

**FIGURE 2:**
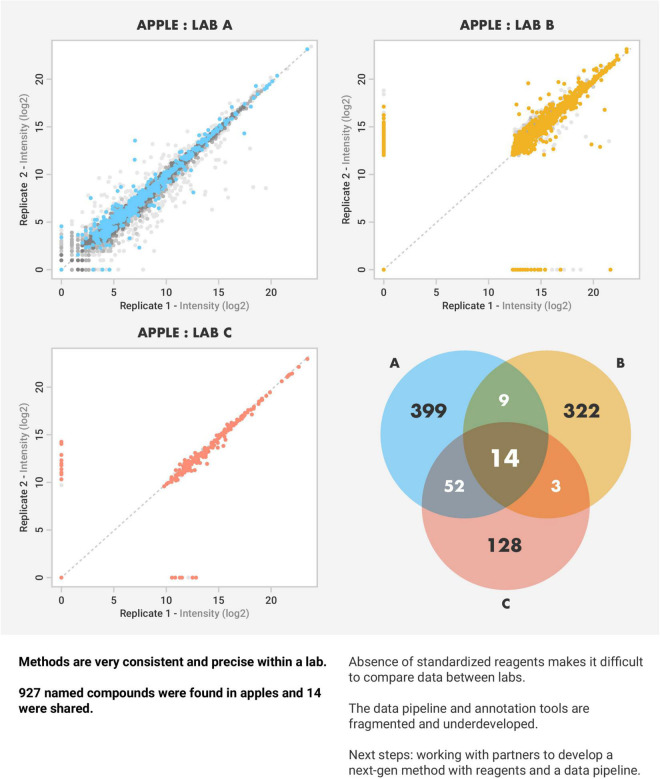
Variability of apple analytes across labs. The Periodic Table of Food Initiative sent identical apple reference sample material to three high-profile laboratories and found tremendous variation in the analytes identified and annotated between the labs. These findings highlight the lack of standardization in food composition analysis while calling for the need for standardized pipelines for analysis and data processing.

Beyond the most commonly measured nutrients, there are limited globally accepted standardized methods to evaluate the multitude of bioactive molecules in food. Labs generally use different protocols for evaluating food biomolecules and focus on identifying and quantifying different sets of biomolecules. The lack of standardized pipelines for data processing results in variation in what biomolecules are identified and quantified. For example, the PTFI sent identical apple reference sample material to three high-profile laboratories and found (Figure 2). The lack of standardized analytical protocols, along with divergent sets of biomolecules measured, are limiting factors to compare data across studies that prevent the identification of global trends in food composition over time (24–26).

### Representation and Quantification Challenges

The planet has abundant edible biodiversity, with some estimates of over 30,000 edible species (27). However, only the most commonly consumed foods are generally represented in food composition databases (approximately 300–400 single-ingredient foods in many food composition databases) due to resource constraints. The FAO/INFOODS Food Composition Database for Biodiversity was developed to broaden the representation of edible biodiversity reported below the species level (i.e., variety/cultivar/breed level) and wild foods in food composition databases (28). Given the impact of socio-ecological factors (i.e., cultivar, soil, climate, growing practices, season, elevation, geography, processing, storage, and cooking) on variability of the presence and quantity of food biomolecules (25), there is a need for food composition databases to provide metadata on these factors as well as update food composition data over time. The Foundation Foods data type of USDA FoodData Central has evolved to focus on the variability of raw foods in the US food supply with metadata associated with foods including genetics, environment, management, and processing (13).

Comprehensive profiling of the broad diversity of food biomolecules is missing from food composition databases (29). Most highlight up to 150 biomolecules of the most well studied macro- and micronutrients in food, which represent less than 1% of the more than 26,000 distinct, definable biochemicals in the scientific literature (29). There are thousands of additional unknown compounds in food based on unidentified spectral data, which collectively with poorly studied identified compounds represent the dark matter of nutrition that may play a powerful role in health (29). A notable percentage of biomolecules in food composition databases are not quantified (29), which limits the utility of these data for informing health and nutrition. The specific concentration of biochemicals can be critical for preventing deleterious health impacts, or providing sensory benefits for consumers.

Further, food composition databases need quantitative data on biomolecules both endogenous (produced through a species’ metabolic and survival processes) and exogenous (natural and synthetic compounds derived from production, postharvest handling, processing, and packaging) to food species. While endogenous food compounds often have the potential for health-promoting effects (15, 16), many exogenous food components can have adverse effects on human and environmental wellbeing. For example, multiple exogenous food components are associated with metabolic diseases, neurodevelopmental disorders, estrogenic activity, and carcinogenicity (17–20).

### Accessibility Challenges

The complexity of food drives accessibility challenges of costly, time-consuming, and low-throughput extraction and analytical methods. Highly sensitive regulatory assays used to populate many national food composition databases are cost-prohibitive. As a result, many lower and middle-income countries maintain and utilize incomplete and/or outdated food composition databases, although some have championed efforts for food composition databases that represent a diversity of their local foods. Further, some national food composition tables are not open-access and/or are only available in the local language with ambiguous codes. With scientific data on more detailed food composition typically staying within peer reviewed journals, only a small fraction of food composition data informs databases. These limitations make it near impossible to use advanced data-mining approaches to discover food compositional patterns and drivers. Additionally, data that are presented in databases are often not in a format for users beyond the research or clinical community.

## State-of-the-Art Omics Technology

Foodomics is driving a knowledge revolution toward a comprehensive understanding of what is in our food, how this composition varies with production and processing factors, and application of detailed food composition knowledge to improve nutrition and health. Here, we highlight three key advances in omics technology enabling unprecedented food composition analysis and application: (i) high-throughput platforms for analysis of a broad range of food molecules; (ii) high-resolution biochemical libraries; and (iii) data integration and machine learning.

### High-Throughput Platforms

State-of-the-art foodomics is based on high-throughput omics platforms (including metabolomics, glycomics, lipidomics, ionomics, and proteomics) to comprehensively profile complex mixtures of biomolecules using mass spectrometry (MS) techniques, nuclear magnetic resonance, and bioinformatics tools (21). The analytical capability of MS, including high-resolution MS instrumentation such as quadrupole time-of-flight and orbitrap systems, coupled with separation instrumentation, enables characterization of the complex components in food at the biomolecule level. More recently, low-cost benchtop instrumentation and software to manage data from complex mixtures are allowing for reengineering and integration of MS methods into cost-effective high-throughput versions to profile food with high sensitivity.

### High-Resolution Biochemical Libraries

High-resolution MS for food composition analysis has led to robust chemical libraries to provide standard reference for identifying biomolecules based on their mass spectra with greater accuracy and confidence. The physical availability of compounds allows us to confirm the presence of a molecule in a sample, while spectral libraries are beginning to allow us to categorize unknown molecules based on chemical similarity, leading to more accurate annotation of unknowns. Emerging open-source high-resolution biochemical libraries offer comprehensive spectral libraries that increase the efficiency of identifying and quantifying thousands of food biomolecules.

### Data Integration and Machine Learning

Advances in data science and informatics are enabling data integration across multiple omics platforms as well as opportunities for untargeted analysis of food components (21, 23). Data integration opportunities are allowing us to make linkages between diverse datasets for addressing critical questions across the food system. Once we can comprehensively profile food using standardized means, we can identify agricultural and production drivers of food composition, while identifying health attributes of food biomolecules. We can further mine integrated datasets on dietary interventions to explore health attributes of food biomolecules and dietary patterns (30, 31).

## Application of Foodomics

Since the emergence of the concept in 2009, foodomics has been applied to create solutions to address global challenges (22, 23). As a data-driven approach, foodomics can be applied in complement to other approaches across the food system for informing evidence-based programs, policies, and practices to improve human and planetary health (Figure 1).

### Nutrition and Health

Foodomics can provide solutions for malnutrition through data on the beneficial and adverse effects of food biomolecules at the biochemical, molecular, and cellular levels (22, 23). The creation of population and individualized diets was considered unapproachable just a few years ago because of the complexities of food, technical challenges, bioavailability and transformation of food biomolecules in the human digestive tract, and the numerous health targets in the human body (23). While still having accessibility challenges to benefit the global community, precision diets are being made possible by identifying foods and dietary patterns on the basis of individual genomes, lifestyles, food preferences, and food access (22).

Data from foodomics can be applied to improve dietary quality by informing dietary guidelines, food procurement, food safety, food labels, product development, food authenticity and traceability, and agricultural guidelines. For example, application of foodomics has proven valuable for identifying dietary biomarkers (32) to elucidate dietary intake to complement traditional dietary assessment methods (33–35) for a more comprehensive understanding of food intake patterns. Governments can include foodomics as part of their food risk assessment (23). Food composition data with associated metadata can be applied to inform food supply and food procurement by enabling enterprises in the food environment to optimize for food composition, price, diversity, and sustainability attributes. Such data can be used to inform purchasing for national nutritional assistance programs.

Foodomics can be applied to develop food products with biomolecules that support healthy and sustainable diets (36) without sacrificing desirable food properties. For example, peptidomic efforts led to the identification of an immunodominant peptide in gluten responsible for inflammation and subsequent symptoms for people with Celiac disease (37), which has enabled efforts to precisely and efficaciously tackle Celiac disease, ranging from engineering varieties of wheat (38) that lack these epitopes to new drugs and supplements that detoxify the epitope in real time within the stomach (39, 40). Another example of the application of foodomics for product development is the recent discovery of a natural product to replace synthetic blue dye #1 with a unique anthocyanin discovered in red cabbage (41).

### Sustainable Diets

Imagine a world where we can use half the land and water for growing nutrient-dense foods by optimizing production for multiple nutritional and environmental targets. Foodomics applied to agriculture can support sustainability through evidence on impacts and mechanisms of abiotic and biotic factors on crop metabolome and proteome toward informing farm- and crop-improvement investments and policies. While health outcomes remain unknown, a meta-analysis demonstrated that organically produced crops and foods have statistically higher concentrations of antioxidant phytochemical concentrations compared to those produced in conventional systems (42). Additionally, foodomics can provide nuanced understanding of food composition in underutilized crops and next-generation crop varieties to support population health while considering multiple sustainability targets. Foodomics applied to the food industry can reduce barriers to enter regulated markets while driving a paradigm shift toward increased availability of affordable functional foods from sustainable agriculture as mainstream in a knowledge-based bioeconomy (43).

Foodomics approaches can provide evidence for the conservation of biocultural diversity associated with Indigenous Peoples’ Food Systems. Efforts led by, or in partnership with, Indigenous communities can apply foodomics to validate biochemical, sensory, and health attributes of Indigenous foods. For example, Lin et al. (44) demonstrated variation of microbiological and biochemical profiles during ripening of Laowo dry-cured ham, an Indigenous fermented food.

## Discussion

There is much to be gained by deepening our knowledge of food composition. Foodomics provides data-driven opportunities to complement existing approaches such as those on dietary quality for advancing nutrition and sustainable diets. Highlighting efforts of PTFI, a participatory effort to create a globally shared foodomics platform, we outline three recommendations to move foodomics forward for people and the planet while addressing unintended consequences.

### Recommendations to Advance Foodomics for Long-Term Translational Impact

#### Global Coordination and Standardization

Foodomics needs to draw from the globally coordinated approach of the Human Genome Project to advance the contribution of food for health. PTFI is developing standardized and democratized protocols to comprehensively evaluate what is in food. This data will be shared in an accessible database that is connected and integrated with existing food composition databases. The success of foodomics requires partnership across sectors and disciplines in ways that respond to evolving needs and opportunities.

#### Integrated Food Systems Approach

Given the complexity of the diet, we need to shift the paradigm away from a reductionist approach, focusing on a limited set of biomolecules. Rather, we need a sustainable food systems approach that evaluates a broad range of what is in food and recognizes the connections of food composition with the environment, socio-economics, equity, and health. PTFI is creating a data platform that brings together socio-ecological metadata associated with foods and health outcomes to understand the complex interactions and trends in food composition.

#### Capacity Strengthening, Inclusion, and Access

For foodomics to realize benefits for societies globally, inclusive and co-creative processes are called for that build on existing knowledge and resources. Food and health outcomes data must be representative and generated by scientists in all countries, as well as shared in ways that are accessible and have meaning to all. PTFI strives to boost foodomics capacity in low- and middle-income countries through laboratory infrastructure development while offering continuing education modules for different users of food composition data.

### Addressing Unintended Consequences of Foodomics

#### Avoiding Access and Benefit Sharing Issues/Biopiracy

Given the reliance of foodomics on genetic resources, it is critical to co-create and operationalize clear access and benefit-sharing statements of genetic resources to protect Indigenous groups and other populations and prevent biopiracy. Foodomics efforts need to abide by the guiding philosophies for genetic resource acquisition set by the International Treaty on Plant Genetic Resources for Food and Agriculture, the Convention on Biodiversity, and the Nagoya Protocol. It is the highest hope that PTFI can act to protect the inherent value of underutilized crops and Indigenous resources from private interests that may seek to benefit unfairly from data.

#### Preventing Biodiversity Loss of Food Species

It is essential for foodomics to acknowledge the conservation status of species and not promote overharvesting. Multiple food species utilized by populations around the world are on the IUCN Red List. These foods may have been historically important to the culture, nutrition, and survival of a population and may continue to be part of cultural identity. Foodomics efforts should not include resources listed as endangered or vulnerable on the IUCN Red List, or listed under CITES. PTFI seeks to promote sustainable use of biodiversity and conservation of habitats that support culturally relevant foods and nutrition.

#### Mitigating Health Disparities

Foodomics approaches must intentionally foster inclusion and justice in food and health systems. Researchers, practitioners, and policy makers need to identify and tackle the technical issues in the application of foodomics that impede equity and exacerbate disparities. The PTFI is developing a health equity framework to guide the application of foodomics in research, programming, practice, and policy.

## Conclusion

Foodomics is leading the way for a data-driven revolution for improving nutritional, health, and environmental targets linked to food. With foodomics data, we have the potential to more precisely define what comprises poor diets as well as healthy and sustainable diets at the biomolecule level. Foodomics is enabling robust determination of environment–diet–health associations. We are at a legacy moment for changing the history of food knowledge. However, without a globally coordinated approach, the potential of foodomics will be stalled. We call for global intersectoral collaboration through PTFI for long-term translational impacts for human and planetary health.

## Data Availability Statement

The original contributions presented in the study are included in the article/supplementary material, further inquiries can be directed to the corresponding author/s.

## Author Contributions

SA, JPa, IE, BG, AJ, VL, AL, TL, CM, NV-M, JPr, CA-S, WS, MR, TS, JS, RS, JT, and SW contributed to the writing, editing, and approval of this perspective. SA, JPa, and BG contributed to the conceptualization of this perspective. All authors contributed to the article and approved the submitted version

## Conflict of Interest

SA was employed by the American Heart Association, Inc. TS and SW was employed by the Verso Biosciences, Inc. The remaining authors declare that the research was conducted in the absence of any commercial or financial relationships that could be construed as a potential conflict of interest.

## Publisher’s Note

All claims expressed in this article are solely those of the authors and do not necessarily represent those of their affiliated organizations, or those of the publisher, the editors and the reviewers. Any product that may be evaluated in this article, or claim that may be made by its manufacturer, is not guaranteed or endorsed by the publisher.
